# Clinical Holistic Medicine: Applied Consciousness-Based Medicine

**DOI:** 10.1100/tsw.2004.8

**Published:** 2004-03-03

**Authors:** Søren Ventegodt, Joav Merrick

**Affiliations:** ^1^ The Quality of Life Research Center, Teglgårdstræde 4-8, DK-1452 Copenhagen K, Denmark; ^2^ National Institute of Child Health and Human Development, Office of the Medical Director, Division for Mental Retardation, Ministry of Social Affairs, Jerusalem and Zusman Child Development Center, Division of Pediatrics and Community Health, Ben Gurion University, Beer-Sheva, Israel

**Keywords:** quality of life, QOL, philosophy, human development, clinical holistic medicine, public health, Denmark, Israel

## Abstract

Consciousness-based medicine is our term for a form of medical treatment that works by direct appeal to the consciousness of the patient, in contrast to modern biomedical treatment where drugs are used to affect body chemistry. With this concept, maybe we are (in a sense) turning back to the “old medicine”, where the family physician was the all-concerned “old country doctor” who knew the child, the siblings, the parents, the family, and the village. In a series of papers on clinical holistic medicine, we would like to present the classic art of healing, where the physician works mostly with his hands, then show how the modern biomedical physician performs with biochemistry, and finally introduce consciousness-based medicine. Some of our questions will be: If you improve your quality of life, will you also improve your health? Will learning more about yourself bring more purpose in your life? Will finding someone to live with in a loving and mutually respectful relationship improve your health? Scientists and thinkers like Antonovsky, Frankl, Maslow, and Jung have pointed to love as a unique way to coherence in life, and thus to biological order and a better health. Several scientific studies have also suggested that patients who focus on improving their quality of life usually will not follow the general statistics for survival, since somehow other factors are at play, which sometimes you will find referred to as “exceptional”.

## INTRODUCTION

Consciousness-based medicine is our term for a form of medical treatment that works by direct appeal to the consciousness of the patient. This is in contrast to modern biomedical treatment, where drugs are usually used to affect body chemistry. In a sense, maybe we are turning back to the “old medicine”, where the family physician was the all-concerned “old country doctor” who came to your home to see your sick child, who you could talk to when social or economic problems became to hard to handle, or who came to be with you when your loved one(s) were dying or had an accident. He knew you and your family and could bring strength, comfort, and support to all.

It is our belief that we all have unlimited hidden resources for healing and curing, which we can call forth when we receive the necessary attention, respect, and care. In the long run, touch, love, and being there for somebody may prove more effective than even the best pills. Perhaps the conscious or unconscious choices that we make every day are decisive for whether we feel well or poorly, healthy or ill. What we experience, decide, anticipate, and feel may affect illness and health far more than we have traditionally assumed. That is what consciousness-based medicine or holistic medicine is about.

## A SERIES ON CLINICAL HOLISTIC MEDICINE

In a new series of papers on clinical holistic medicine, we would like to present the classic art of healing, where the physician works mostly with his hands. Then we will go on to show how the modern biomedical physician performs small miracles with biochemistry. Finally, we will introduce the main topic of this series, consciousness-based medicine: Will improving your quality of life also improve your health? Will learning more about yourself bring more purpose in your life? Will finding someone to live with in a loving and mutually respectful relationship improve your health?

The case stories mainly concern patients who remain chronically ill despite conventional medical treatment and often also despite alternative treatment. We wish to show readers how consciousness-based medicine, by supporting personal development, can help patients to tackle conditions hitherto out of therapeutic reach: chronic pain, rheumatoid arthritis, cancer, heart conditions, psychoses, or dementia.

In our perspective, physical and mental disorders and complaints are often symptoms of a mistaken basic attitude to life. Obviously disorders and complaints may also have different causes, such as purely genetic disorders like muscular atrophy and purely traumatic illnesses like physical injuries caused by accidents, but general practitioners rarely see those in surgery or clinic. They affect a few percent of the population, represent a large cost to the health system, and are usually managed by specialist units at major hospitals or medical centers[1,2].

The more common complaints that we have seen and studied in patients may have hidden genetic or traumatic aspects. First of all, people seem to suffer mostly from complaints caused by their inability to understand themselves, life, or how to relate to others. It is almost as if sick people do not fulfill their destiny in life. Instead, they suppress and fail themselves. Deep down in our existence, we have great hidden resources that are best described in words like wisdom, joy, and energy of life. Once we uncover these resources in ourselves and rely on them more, we slowly change from being dissatisfied, sad, and ill to being far more alive, healthy, and happy.

## CONSCIOUSNESS-BASED MEDICINE AND QUALITY OF LIFE

Consciousness-based medicine is about improving the quality of life through personal development. A few studies in particular seem to indicate that survival in cancer and cardiovascular disorders can be drastically improved or prolonged by a targeted improvement of life[3,4]. Other diseases, like management of autoimmune diseases such as juvenile diabetes (type 1) could probably also be promoted by tackling the patient's inner conflicts, when the first symptoms appear. It is important to underline that hundreds of scientific studies have shown a statistical correlation between health and dimensions like love, intimacy, good relationships, positive philosophy of life, and constructive life strategy[5]. What we need now are suggestions for cures that provide efficient results, which can be scientifically documented with standard protocols or with a method appropriate for consciousness-based medicine[6]. We aim to suggest twenty new holistic cures to be scientifically tested in the near future and with our series of papers on clinical holistic medicine, to accelerate the important development of an evidence-based, consciousness-based medicine, a development that in the end can give the physician another toolbox, more effective and cheaper for the patient, than the pharmaceutical toolbox he has today.

It is evident that we have full confidence in consciousness-based medicine, and we hope that the case histories in this series, which come from our clinical experiences and with qualitative documentation, will confer that confidence on our readers. Our basic perspective in life is that once we humans understand what a mystery we present and what energies we command, and once we take in how we connect to the universe surrounding us, we can also acknowledge our freedom to recreate ourselves as good or evil, healthy or sick, strong or weak.

This does not render our lives free of problems; quite the contrary. We are created with a will to do things, and this is likely to entail problems. The good life is intense and vibrant for better or for worse. The intense emotional pain of the will of doing should be balanced by the intense joy and pleasure of existing, being alive and conscious. That is the great art of living, which the holistic physician should share with his patients.

## LOVE AND TENDER CARE

The articles that soon will appear in this journal also have some unusual cases stories, among them Mia, Anna, and Helena, who suffered from serious injuries to their soul and through treatment and intervention were helped back on track again with an intervention that consisted of love and care. Scientists and thinkers like Antonovsky[7], Frankl[8], Maslow[9], and Jung[10,11] have pointed to love as a unique way to coherence in life, and thus to biological order and a better health; these case histories seem to support that idea.

On the other hand, we want to caution our readers to the fact that we believe all disorders should be investigated and diagnosed. Although the authors believe that improving the quality of life will often be the best and most effective way of preventing and treating illness, efforts to improve the quality of life should be seen as a supplement to conventional medical treatment and not as a replacement for it. We would encourage anyone suffering from or suspecting illness to seek complete medical assessment and subsequently decide what treatment would be desirable for the disease in question based on current experience with different treatment options. Several scientific studies suggest though that patients focused on improving their quality of life usually do not follow the general statistics for survival, since somehow other factors are at play, which sometimes you will find referred to as "exceptional".

## ACKNOWLEDGMENTS

These studies were supported by grants from IMK Almene Fond. Our research in quality of life has been approved by the Copenhagen Scientific Ethical Committee under number (KF)V.100.2123/91.

## BIOSKETCHES

**Søren Ventegodt, MD,** is the Director of the Quality of Life Research Center in Copenhagen, Denmark. He is also responsible for a Research Clinic for Holistic Medicine in Copenhagen and is a popular speaker throughout Scandinavia. He has published numerous scientific or popular articles and a number of books on holistic medicine, quality of life, and quality of working life. His most important scientific contributions are the comprehensive SEQOL questionnaire, the very short QoL5 questionnaire, the integrated QOL theory, the holistic process theory, the life mission theory, and the Danish Quality of Life Research Survey, 1991–94 in cooperation with the University Hospital of Copenhagen and the late pediatric professor Bengt Zachau-Christiansen. E-mail: ventegodt@livskvalitet.org. Website: www.livskvalitet.org

**Joav Merrick, MD, DMSc,** is Professor of Child Health and Human Development affiliated with the Zusman Child Development Center, Division of Pediatrics and Community Health at the Ben Gurion University, Beer-Sheva, Israel; the Medical Director of the Division for Mental Retardation, Ministry of Social Affairs, Jerusalem; and the Founder and Director of the National Institute of Child Health and Human Development. He has numerous publications in the field of child and human development, rehabilitation, intellectual disability, disability, health, welfare, abuse, advocacy, quality of life, and prevention. Dr. Merrick received the Peter Sabroe Child Award for outstanding work on behalf of Danish Children in 1985 and the International LEGO-Prize ("The Children's Nobel Prize") for an extraordinary contribution towards improvement in child welfare and well being in 1987. E-mail: jmerrick@internet-zahav.net. Website: www.nichd-israel.com
